# Single dose effect of diazinon on biochemical parameters in testis tissue of adult rats and the protective effect of vitamin E

**Published:** 2014-11

**Authors:** Fatemeh Rahimi Anbarkeh, Mohammad Reza Nikravesh, Mehdi Jalali, Hamid Reza Sadeghnia, Zinat Sargazi, Leila Mohammdzadeh

**Affiliations:** 1 *Department of Anatomy and cell Biology, School of Medicine, Mashhad University of Medical Sciences, Mashhad, Iran.*; 2 *Department of Pharmacology, Faculty of Medicine, Mashhad University of Medical Sciences, Mashhad, Iran.*; 3 *Department of Pharmacodynamics and Toxicology, Faculty of Pharmacy, Mashhad University of Medical Sciences, Mashhad, Iran.*

**Keywords:** *Diazinon*, *Vitamin E*, *Malondialdehyde*, *Glutathione*, *Testis*

## Abstract

**Background:**

Diazinon (DZN) is an organophosphate pesticide that widely used for agricultural pest control all over the world. DZN affects target organs including reproductive system by inhibiting the activity of acetylcholinesterase and inducing oxidative stress. Vitamin E (α-tocopherol) is a strong antioxidant which inhibits free radicals, and probably can reduce lipid perxidation effectively in biological systems.

**Objective:**

The present study, aimed to evaluate the effects of DZN on malondialdehyde (MDA) and glutathione (GSH) levels in testis of rats and protective effect of vitamin E.

**Materials and Methods:**

In this experimental study, thirty adult male Wistar rats (200-250 gr) were divided into 5 groups (n= 6): control group (did not receive any material), sham group (received only pure olive oil), experimental group 1 (DZN, 60 mg/kg), experimental group 2 (Vit E, 200 mg/kg) and experimental group 3 (DZN+Vit E, with the same dose). All groups were sacrificed after 6 weeks and right testis was used to measure the MDA and GSH levels. The amount of MDA was determined by the thiobarbituric acid assay and 5, 5-Dithio-bis (2nitrobenzoic acid) DTNB-recycling protocol was used for GSH assay.

**Results:**

The results showed that DZN increased MDA level (p<0.001) and reduced GSH level (p<0.001). Administration of DZN plus vitamin E decreased the MDA level (p<0.001) and increased GSH level (p=0.001).

**Conclusion:**

DZN induced lipid peroxidation in the testis of rats. Vitamin E by its antioxidant activity was able to improve the toxic effect of DZN.

## Introduction

Organophosphate (OP) pesticides are a main group of chemical insecticides which their use has been raising worldwide, especially in developing countries. DZN (o,o-diethyl-o-[2-isopropyl- 6- methyl- 4- pyrimidinyl] phosphorothioate) is an OP pesticide which is extensively used to control households insects and vegetable crops. OP residues have been observed in soil, water, vegetables and other foods (1). It affects various organs of the body including liver and muscle, kidneys, pancreas, spleen, brain and reproductive systems (2-6). Researches have shown that several factors such as duration and concentration of DZN, are affecting its toxicity (7, 8). Some studies have shown that DZN was capable of inducing some biochemical alterations in the ovaries and testes, also it can adversely affect the reproductive function by reducing the number of Leydig cells and the mass of testis and changing the gonadotropin levels (9). 

DZN inhibit acetylcholinesterase (AChE) activity in the target tissues, which is the most important action of DZN compound. AChE is an enzyme that catalyzes acetylcholine and prevents its accumulation at cholinergic synapses (1, 3). Oxidative stress is other mechanism that isinvolved in DZN toxicity which creates reactive oxygen species (ROS) and changes the enzymatic activities associated with antioxidant defense mechanisms in the body (10, 11). A variety of enzymatic and nonenzymative processes can generate ROS in mammalian cell. Depending on the nature of the ROS species, some are highly toxic and rapidly detoxified by enzymatic and non-enzymatic processes (12). 

The cellular structure, motility, survival, semen parameters, metabolic functions of the sperm and capacity for sperm-oocyte fusion can be impaired as a result of lipid peroxidation caused by ROS. MDA which is a stable lipid peroxidation product can be used as a marker of oxidative stress (13, 14). Thiol groups are sensitive to oxidative damage, and when oxidative damage occurs, these groups will be able to scavenge the free radicals. GSH is one of the thiol groups and is also an essential component to the body's natural defense system. Thus, the severity of induced oxidative stress by DZN can be determined by measuring these groups (11, 15-17). 

On the other hand, considering the wide use of DZN, one of the ways to deal with its toxicity, is antioxidants. There are two forms of antioxidant; enzymatic and nonenzymatic (13). Vitamin E is known as a fat soluble non-enzymatic antioxidant which interrupts release of lipid peroxidation in the plasma membrane and thus maintains the integrity of the membrane. Many studies had shown that Vitamin E inhibit oxidative stress and lipid peroxidation induced by OP pesticides in experimental animals (10, 18, 19).

In reproductive system, vitamin E prevent the damaging effects of active oxygen radicals on spermatogenesis and sperm health and therefore reduce testicular oxidative stress (1, 20). According to the mentioned evidences, this study was designed to determine glutathione and malondialdehyde, as lipid peroxidation biomarkers in testis of male rats and to assess whether these effects can be prevented or improved by vitamin E.

## Materials and methods

**Animals**

The present study was performed in pharmacology laboratory of Pharmacy Faculty of Mashhad University of Medical Sciences. In this experimental study, thirty adult male Wistar rats (200-250 g) were divided into 5 groups (n= 6): control group (did not receive any material), sham group (received only pure olive oil), experimental group 1 (DZN, 60 mg/kg), experimental group 2 (Vit E, 200 mg/kg) and experimental group 3 (DZN +Vit E, with the same dose) (21). 

Technical DZN with lethal dose of 100 (LD50) was used and based on that, does of 60 mg/kg was determined. The olive oil was used as a solvent; the stock solution was made freshly by DZN with density of 60 mg/ml in olive oil. DZN and solvent were injected intraperitoneally (IP) and vitamin E was administrated orally by gavage. All animals were sacrificed after 6 weeks and right testis was used to measure the MDA and GSH levels. The rats were fed a standard chow and water ad libitum, and exposed to a 12-hour light/dark cycle, at a temperature of 22^o^C. All the experimental protocols were approved by the Ethical Committee of Mashhad University of Medical Science.

**Chemicals**

For this study, technical DZN 98% was purchased from Ariashimi Company. DZN was dissolved in olive oil. Malondialdehyde tetrabutylammonium, reduced glutathione (GSH), DTNB [5, 5′ di thiobis-(2-nitrobenzoic acid)] and vitamin E (α-tocopherol acetate) were purchased from Sigma.

**Lipid peroxidation assay**

The amount of lipid peroxidation was assessed through the measurement of MDA levels in testis tissues. MDA reacts with thiobarbituric acid (TBA) and produce a pink colored complex which has the maximum absorbance at 532 nm. To start working, 3 ml phosphoric acid (1%) and 1 ml TBA (0.6%) were added to 10% homogenated tissue in KCl, and then the compound were heated for 45 min in a boiling water bath. After cooling the compound, 4 ml of n-butanol was added to it and vortex-mixed for 1 min followed by centrifugation at 3000 gr for 10 min. 

Then, the organic layers were removed and transferred to fresh tubes and the absorbance level was read at 532 nm (22). A calibration curve was constructed using malondialdehyde tetrabutylammonium. To determine the malondialdehyde density, at first the malondialdehyde density in testis tissue by n mol/ml was calculated based on equation of the line obtained by malondialdehyde standard curve, then obtained number was transferred to nmol/g tissue.

**Reduced glutathione assay**

GSH was evaluated in testis tissue by the Moron *et al* method (23). The basis of the work was the formation of yellow color after adding DTNB [5,5’ di thiobis-(2-nitrobenzoic acid)] to compounds containing sulfhydryl groups. For this purpose, 300 µl of homogenates were blended with 300 µl of 10% tricolor acetic acid (TCA) and vortexed. After centrifugation at 2500 g for 10 min, the upper layers were removed and mixed with reaction mixtures containing 2 ml phosphate buffer (pH: 8) and 500 µl of DTNB 4% reagent was prepared and mixed with citrate sodium 10%. After 10 min, the absorbance was evaluated at 412 nm using a spectrophotometer (Jenway 6105 uv/vis, UK). At the end, the amount of GSH was determined based on a standard curve drawn with commercially available GSH. The GSH levels were expressed as nmol/g tissue

**Statistical analysis**

Results are expressed as mean±SD. Data were analyzed using SPSS software. Statistical analysis was performed with one way ANOVA followed by Tukey post hoc test to compare the differences between means. Differences were considered statistically significant when p<0.05.

## Results

**Effect of Vit E on** **testis** **lipid peroxidation induced by DZN**

The mean±SD of lipid peroxidation, was determined with MDA assay. Comparison of means of MDA level in different treatment groups had shown no significant differences between control, sham and treatment groups 2 (control vs. sham p=0.98 and control vs. experimental group 2 p=0.90). Exposure to DZN in experimental group 1 significantly increased MDA level compared to control group and other groups (p<0.001). The mean±SD of MDA level experimental group 1 and 3 were 179.55±3.2 and 146.58±1.5 nmol/g tissue respectively. Therefore simultaneous administration of DZN with Vit E in experimental group 3, decreased MDA level in testis tissue when compared to experimental group 1(p<0.001). Experimental group 3 had shown significant differences compared to control, sham and experimental groups 2 (p<0.001) (Table I).

**Effect of Vit E on GSH content in testis** **tissue following exposure to DZN**

The means of glutathione and malondialdehyde levels in different groups of adult male rats are demonstrated in table II, GSH content had shown no significant differences between control, sham and experimental groups 2 (control vs. sham p=0.17 and control vs. experimental group 2 p=0.98). The mean±SD of GSH for experimental group 1 was 4493.681±16.32 nmol/g tissue. A significant decrease in GSH content of testis was observed following exposure to DZN in experimental group 1 in comparison with control, sham and experimental groups 2 (p<0.001). The mean±SD of GSH for experimental group 3 was 5831.297±15.65 nmol/g tissue, therefore Vit E in experimental group 3 significantly increase the GSH level in testis tissue compared to experimental group 1 (p=0.001). In addition experimental group 3 had shown significant differences compared to control, sham and experimental groups 2 (p<0.001).

**Table I:** Mean± SD of malondialdehyde among various groups in testis

**Group**	**Malondialdehyde** *	**p-value** **vs. control group**	**p-value** **vs. DZ group**
Control	113.71 ± 3.5^b^		<0.001
Sham (Oil 1 ml/kg)	115.83 ± 2.3^b^	0.98	<0.001
Exp.1 (DZ 60 mg/kg)	179.55 ± 3.2^d^	<0.001	<0.001
Exp.2 (Vit E 200 mg/kg)	110.25 ±3.4^b^	0.90	<0.001
Exp.3 (DZ 60 mg/kg+Vit 200 mg/kg)	140.78 ± 1.7^c^	<0.001	<0.001

* Values with small different letters in columns shown that are significantly different and similar letters are not significantly different (p<0.05) in various groups.

**Table II:** Mean± SD of glutathione among various groups in testis

**Group**	**Glutathione** *	**p-value** **vs. control group**	**p-value** **vs. DZ group**
Control	8898.636 ± 24.01^c^		<0.001
Sham (Oil 1 ml/kg)	8440.769 ± 15.78^c^	0.17	<0.001
Exp.1 (DZ 60 mg/kg)	4493.681± 16.32^a^	<0.001	<0.001
Exp.2 (Vit E 200 mg/kg)	8964.213 ± 19.21^c^	0. 98	<0.001
Exp.3 (DZ 60 mg/kg+Vit 200 mg/kg)	5831.297 ± 15.65^b^	0.001	<0.001

* Values with small different letters in columns shown that are significantly different and similar letters are not significantly different (p<0.05) in various groups.

## Discussion

DZN is an organophosphate pesticide which is extensively used to control households insects and vegetable crops. Indiscriminate use of pesticides including DZN has been one of the problems of current society, especially in the major centers of agriculture and cause negative effects on fertility. DZN by making changes in the DNA or in proteins bound can damage testis tissue and create mutations in spermatogonia which eventually lead to changes in the sperm (14, 24). The present study investigated the protective effect of vitamin E against diazinon -induced toxicity in the testis of rat. For this purpose, rats were treated with DZN (60 mg/kg) and vitamin E (200 mg/kg) for 6 weeks. 

Finally, for determining the toxicity both glutathione and malondialdehyde levels were measured. Lipid peroxidation, destroy the unity of cellular membranes and lead to the leakage of cytoplasmic enzymes (25). In the present study DZN in experimental group1 significantly increased MDA level as compared to control group which can be associated with leakage of cytoplasmic enzymes. But administrating vitamin E plus DZN in experimental group 3 significantly decreased MDA levels. One of the mechanisms of DZN toxicity is inducing oxidative stress and producing of ROS. Since DZN was used in experimental groups 1, we observed the highest MDA level in experimental groups 1. In the present study, the amounts of GSH reduced in experimental group 1. Therefore we observed reduced antioxidant activity in testis tissue following the use of OPs and producing of ROS. Also, in this study, the simultaneous use of DZN plus vitamin E in experimental groups3 increased the GSH level.

Akturk *et al* showed that DZN increased MDA level and increased superoxide dismutase and catalase activities in the heart. The simultaneous use of DZN plus vitamins C and E reduced the levels of MDA and superoxide dismutase (16). In another study, malathion induced testicular toxicity, the toxicity was reduced by vitamins E and C, but not resolved completely (1). Other studies have shown that DZN increase MDA level in blood, myocardial cells, liver, muscle and brain (2, 7, 8, 10, 18, 26). The results of present study and similar studies indicated the induction of lipid perxidation and consequently increase in malondialdehyde level by DZN.

DZN in different concentrations induces oxidative stress and therefore induction of ROS can lead to oxidative injuries of important cellular macromolecules such as lipids, proteins, and nucleic acids (27). GSH plays a role as an antioxidant in the body and its presence is necessary for protection against free radicals (10). In the study of Isik *el al* administration of methyl parathion and DZN to fish reduced the glutathione level in liver tissue (11). Ahmadi *et al* showed that DZN increased GST enzyme activity in spleen tissue and increased consumption of GSH, which indicates the increase of body's defense against toxin, and rapid excretion of it (4). The reduced glutathione reservoir in the body and reduced antioxidant activity in many tissues following the use of OPs were observed (17, 28). 

The administration of DZN in other studies, caused reduction in GSH level in the liver (10, 29). Shah *et al* reported that the use of DZN significantly increased lipid peroxidation in a dose dependent manner in renal tissue and with increasing DZN dose, a greater increase in MDA level was observed. they also demonstrated that DZN treatment decreased renal glutathione, and reduced the activities of antioxidant enzymes including the enzymes involved in glutathione metabolism and produced oxidants in large amount with concomitant renal damage, which all of them are involved in the cascade of events leading to DZN-mediated renal oxidative damage and toxicity (3). 

Free radicals and reactive oxygen species play an important role in reducing the quantity and quality of semen and cause the loss of vital sperm activities (30, 31). Therefore evidences show that glutathione through reaction with oxygen-containing functional groups like free radicals provide its protective role; this is the reason for reducing this antioxidant in toxicity caused by DZN. Faced with DZN, enzymatic and non-enzymatic antioxidant systems for protecting the body against ROS are activated (3, 32). Vitamin E, is a lipid-soluble vitamin that is involved in scavenging of free radicals (30). 

In this study, in experimental group 3 (DZN plus vitamin E), vitamin E reduced the malondialdehyde level and increased glutathione level as compared with experimental group 1, that is due to inhibitory effects of vitamin E on oxidative stress and tissue lipid peroxidation. Other studies showed that vitamin E, as a strong antioxidant, can protect against the damaging effects on reproductive system (20, 33, 34). 

Sameni *et al* showed that cyclosporine causes severe degenerative changes in the testes. Treatment with vitamin E decreased these changes, and after using it, testicular structure and spermatogenesis showed conditions close to normal situation (19). In the Yilmaz study, oxidative stress was involved in brain toxicity induced by DZN and the results showed that compounds of vitamins C and E may have a protective effect (5). Sutcu *et al* reported that DZN administration increased lipid peroxidation in rat erythrocytes and combinations of vitamins C and E can help to reduce it (35). 

According to the biochemical effects of DZN in rat testis (reducing GSH content and increasing MDA level), there is a risk of cytotoxicity in the farmers and other people who are in contact with these compounds. Vitamin E can be considered as a potential therapeutic agent for testicular toxicity caused by reducing MDA level and increasing GSH content. Therefore, it is necessary to prevent toxins entry to the body which is causing gonadal dysfunction.

## Note

***This article extracted from M.Sc. thesis. (Fahime Rahimi Anbarkeh)***
